# Subchronic effects of HgCl_2_ on cognitive function and central inflammation in type 2 diabetic rats: involvement of BDNF and acetylcholinesterase

**DOI:** 10.3389/ftox.2025.1610720

**Published:** 2025-07-14

**Authors:** Douae Benloughmari, Samir Bikri, Meriam El Aboubi, Fatima-Zahra Yassif, Youssef Aboussaleh

**Affiliations:** ^1^ Faculty of Sciences, Laboratory of Biology and Health, Ibn Tofail University, Kenitra, Morocco; ^2^ Higher School of Technology, Ibn Tofail University, Kenitra, Morocco; ^3^ Laboratory of Natural Resources and Sustainable Development, Biology Department, Ibn Tofail University, Faculty of Sciences, Kenitra, Morocco

**Keywords:** type 2 diabetes, mercury chloride, neuroinflammation, learning and memory, BDNF, acetylcholinesterase

## Abstract

**Introduction:**

Type 2 diabetes mellitus (T2DM) is a major global health concern frequently related with chronic low-grade inflammation and a spectrum of cognitive impairments, including deficits in learning and memory. Mercury chloride (HgCl_2_), a widespread environmental pollutant, is recognized for its neurotoxic properties and its capacity to trigger inflammatory responses, particularly in patients with metabolic disorders such as T2DM.

**Aim:**

This study aimed to evaluate the subchronic effects of HgCl_2_ on cognitive performance and neuroinflammation in a rat model of T2DM, with a particular focus on the roles of BDNF and acetylcholinesterase (AChE).

**Materials and methods:**

The experimental design included four groups: control, HgCl_2_-treated, diabetic, and diabetic rats treated with HgCl_2_. T2DM was induced by intraperitoneal injections of streptozotocin (STZ) and nicotinamide (NA). Rats in the HgCl_2_-exposed groups received an oral dose of 0.375 mg/kg/day for 45 consecutive days. Cognitive performance was assessed using behavioral tests targeting spatial learning, recognition memory, and working memory. Additionally, hippocampal and prefrontal cortex (PFC) levels of TNF-α, IL-6, BDNF, and AChE activity were measured to evaluate neuroinflammatory and neurotoxic responses.

**Results:**

The findings revealed a significant increase in fasting blood glucose levels in both diabetic and HgCl_2_-treated diabetic groups compared to controls (P < 0.001). Moreover, HgCl_2_ administration in diabetic rats led to a more pronounced impairment in cognitive functions compared to untreated diabetic rats (P < 0.05). These deficits were associated with enhanced neuroinflammatory markers (TNF-α and IL-6), decreased AChE activity, and reduced BDNF expression in the PFC and hippocampus (P < 0.05).

**Conclusion:**

Overall, these results highlight the synergistic impact of hyperglycemia and HgCl_2_ exposure in exacerbating neuroinflammation and cognitive decline, suggesting a critical interaction between metabolic and environmental neurotoxic factors.

## 1 Introduction

Type 2 diabetes (T2D) is a chronic metabolic disorder characterized by insulin resistance, hyperglycemia, and β-cell dysfunction (So and Accili, 2023), leading to widespread systemic complications, including cognitive impairment (CI) (Liu, et al., 2025; Srikanth et al., 2020). Cognitive dysfunction in T2D is increasingly recognized as a significant complication, contributing to reduced quality of life and increased healthcare burden (Srikanth et al., 2020). Diabetes-related CI results from complex mechanisms affecting brain function; hyperglycemia disrupts cerebral glucose metabolism by altering key pathways such as glucose transport, glycolysis, the pentose phosphate pathwa, and the tricarboxylic acid cycle (Zhang et al., 2023). These metabolic disturbances lead to reduced ATP production, increased oxidative stress and inflammation, and impaired neurotransmitter synthesis, ultimately disrupting synaptic plasticity and contributing to neuronal damage and cognitive decline (Zhang et al., 2023). Additionally, the presence of metabolic syndrome, often associated with T2D, further exacerbates neurocognitive deficits, impairing attention, processing speed, memory, and executive function (Sánchez-Ortí et al., 2022). Neuroimaging studies have revealed structural and functional brain changes, such as cortical thinning, gray-matter atrophy, and white-matter hyperintensities, all of which correlate with CI in diabetic individuals (Moran et al., 2022). Given the significant impact of CI in diabetes, stemming from complex metabolic and vascular disturbances, there is an urgent need for early detection and multifaceted interventions to improve patient outcomes and preserve cognitive function. (Yu et al., 2025). Chronic hyperglycemia plays a pivotal role in the pathophysiology of diabetes-induced CI by promoting neuroinflammation (Yu et al., 2025). Persistent elevated blood glucose levels induce oxidative stress and the release of pro-inflammatory cytokines, such as IL-1β and TNF-α, which contribute to neuronal damage and cognitive deficits (Yao et al., 2024). The activation of neuroinflammatory pathways in T2D has been shown to compromise the blood-brain barrier, leading to increased permeability and neurotoxic accumulation (Yao et al., 2024). Moreover, T2D is closely linked to neurodegenerative diseases such as Alzheimer’s disease through shared mechanisms such as increased inflammation and impaired glucose homeostasis. In T2D, the enzyme Beta-Secretase 1, which plays a key role in the production of amyloid-beta (Aβ) in Alzheimer’s disease, also contributes to metabolic dysfunction; BACE1 activation leads to increased neuroinflammation and impaired insulin signaling, while simultaneously promoting the accumulation of protein tyrosine phosphatase 1B which disrupts insulin signaling and exacerbates glucose homeostasis dysfunction, further linking metabolic dysregulation with neurodegenerative processes. (Franklin et al., 2025). Emerging evidence highlights that hyperglycemia-induced neuroinflammation also involves microglial activation and metabolic reprogramming via the ChREBP/HIF-1α signaling pathway, which promotes glycolysis and sustains an inflammatory state, further contributing to CI (Yao et al., 2024).

Exposure to environmental neurotoxins, such as mercuric chloride (HgCl_2_), has been identified as a significant contributor to neuroinflammation and cognitive dysfunction (Obeng-Gyasi and Obeng-Gyasi, 2024; Althomali et al., 2024). Mercury exposure disrupts pancreatic beta cells by inducing inflammation, which leads to calcium depletion and protein misfolding in the endoplasmic reticulum (ER), impairing insulin secretion. In response, the unfolded protein response is activated to restore ER homeostasis; however, if stress persists, apoptotic pathways involving p38 MAPK, PI3K, JNK, and ROS production are triggered. These results in β-cell death through mitochondrial and ER-mediated apoptosis, ultimately reducing insulin levels and causing hyperglycemia, contributing to diabetes complications (Yuliana et al., 2024). Moreover, studies have demonstrated that HgCl_2_ exposure leads to increased levels of pro-inflammatory cytokines, resulting in neuronal damage and CI (Obeng-Gyasi and Obeng-Gyasi, 2024). For instance, research indicates that HgCl_2_ exposure results in increased levels of pro-inflammatory cytokines, contributing to neuronal damage and CI (Obeng-Gyasi and Obeng-Gyasi, 2024). While direct studies on HgCl_2_’s impact in the context of T2D are limited, the shared pathways of inflammation suggest a potential link between HgCl_2_ exposure and exacerbated cognitive dysfunction in T2D patients (Obeng-Gyasi and Obeng-Gyasi, 2024).

Mercury toxicity also contributes to cholinergic dysfunction, as it decreases acetylcholine (ACh) levels by impairing its binding in the cerebral cortex (Althobaiti, 2025). Additionally, choline acetyltransferase (ChAT), a key enzyme responsible for ACh synthesis, is inhibited by mercury exposure, further exacerbating ACh deficiency and potentially worsening CI (Althobaiti, 2025). The disruption of cholinergic signaling is further compounded by the role of acetylcholinesterase (AChE), an enzyme responsible for ACh hydrolysis at synaptic clefts. Under normal conditions, AChE activity is essential for regulating neurotransmission; however, its dysregulation is strongly linked to cognitive deficits. Excessive AChE activity contributes to cholinergic dysfunction by excessively breaking down ACh, leading to impaired synaptic plasticity, learning, and memory, particularly in key brain regions such as the hippocampus and cortex (Marucci et al., 2021). Furthermore, an imbalance between AChE and butyrylcholinesterase (BuChE) activity has been observed, further exacerbating cognitive dysfunction. In addition to its enzymatic role, AChE interacts with amyloid precursor protein (APP), promoting amyloid-beta (Aβ) aggregation, which accelerates neurodegeneration and worsens cognitive decline (Chen et al., 2022). These disruptions in neurotransmitter function, alongside the reduced release of brain-derived neurotrophic factor (BDNF) in diabetes mellitus, further amplify the risk of CI in affected individuals (Ahmad et al., 2022). BDNF is a key neurotrophin essential for neuronal differentiation, survival, and synaptic plasticity, playing a pivotal role in memory and learning (Bikri et al., 2024), It is primarily expressed in brain regions responsible for higher cognitive functions, including the hippocampus, cortex, and basal forebrain, where it contributes to synapse formation and neurotransmission (Nemati et al., 2025). Notably, BDNF is closely linked to cognitive disorders, with its signaling pathway associated with reduced apoptosis in hippocampal neurons and protection against hippocampal atrophy (Peritore et al., 2020). Furthermore, disruptions in the BDNF-cAMP response element-binding protein (CREB) signaling pathway have been implicated in neurodegenerative diseases, where synaptic loss and memory deficits are prominent consequences of BDNF depletion (Bikri et al., 2024). Upon binding to its receptor, BDNF activates CREB, a critical pathway for the expression of genes necessary for neuronal survival in the hippocampus (Chen, et al., 2024). Conversely, increased BDNF expression has been shown to enhance memory function and provide neuroprotection, reinforcing its vital role in maintaining cognitive health (Bikri et al., 2024). Given its significance in neuronal plasticity and cognitive resilience, alterations in BDNF signaling represent a key factor in the pathology of various neurological disorders (Caviedes et al., 2017).

Considering the critical involevoment of both AChE and BDNF in cognitive function, this dysregulation may serve as mechanistic link between HgCl_2_ neurotoxicity and T2D-related neurodegeneration. The neurotoxic effects of HgCl_2_ in diabetic models remain insufficiently explored, yet its impact on neuroinflammatory pathways suggests a potential interaction between heavy metal exposure and T2D-related cognitive dysfunction. Given the shared mechanisms of neuroinflammation, and metabolic dysregulation in both T2D and HgCl_2_ toxicity, investigating their combined effects on cognitive function is of paramount importance. This study aims to evaluate the subchronic effects of HgCl_2_ on cognitive function and central inflammation in a rat model of T2D, with a specific focus on the involvement of BDNF and AChE. Understanding these interactions could provide novel insights into the environmental factors contributing to T2D-related neurodegeneration and inform potential therapeutic strategies.

## 2 Materiel and methods

### 2.1 Animals

Adult Wistar rats weighing 230–250 g, utilized in this study, were procured from the central animal facility of the Faculty of Science in Kenitra, Morocco. The animals were housed in compliance with standardized guidelines, and all experimental procedures adhered to the ethical standards established by the institution’s ethics committee. The rats were individually accommodated in plexiglass cages and acclimatized for 1 week in a controlled experimental environment. Throughout the acclimatization period, the rats had unrestricted access to food and water.

### 2.2 Experimental induction of diabetes

Type 2 diabetes mellitus (T2DM) in rats was induced using the streptozotocin-nicotinamide (STZ-NA) diabetic animal model, as described by Samir et al. (2022). Following an overnight fast, the rats were intraperitoneally injected with 65 mg/kg of STZ (Sigma, St. Louis, MO, United States) 15 min after the administration of NA (110 mg/kg). Twelve hours after the STZ-NA injection, the animals were provided with a 5% aqueous glucose solution. To confirm hyperglycemia, blood glucose levels were measured 3 days STZ-NA post-injection. Rats exhibiting blood glucose levels ≥250 mg/dL were selected for inclusion in this study.

### 2.3 Experimental design: animal grouping and treatment

**FIGURE 1:**
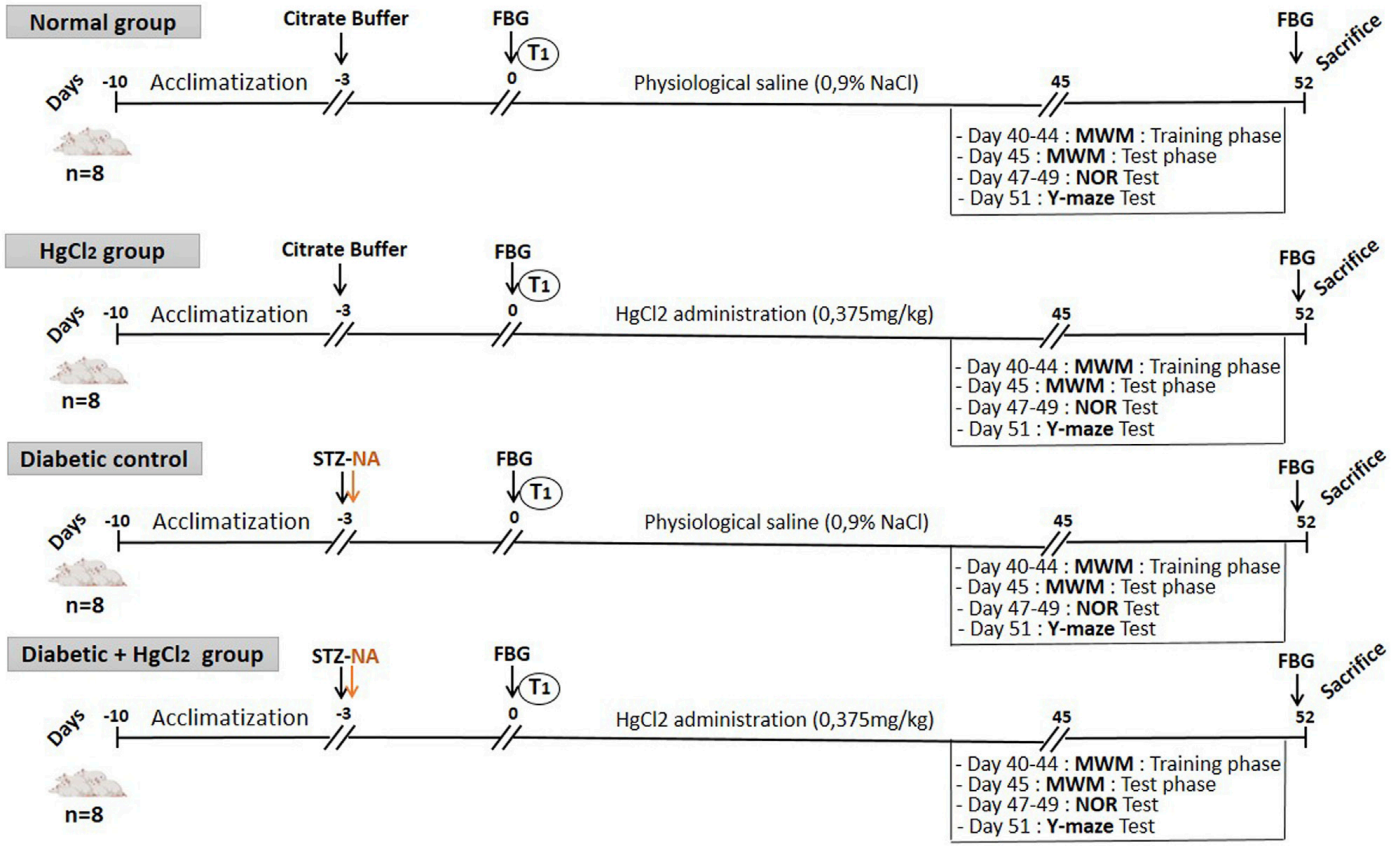
Experimental design: animal grouping and treatment.

In this investigation, rats were assigned to four experimental groups: a non-diabetic control, a diabetic control, a diabetic cohort administered HgCl_2_, and a non-diabetic cohort administered HgCl_2_. The treatment groups (diabetic and non-diabetic) were orally administered HgCl_2_ once daily at 0.375 mg/kg for 45 days, consistent with methodologies outlined by Teixeira et al. (40). Behavioral evaluations, including the Morris Water Maze (Day 40), Novel Object Recognition (day 47) and Y-Maze (day 51) tests, were performed to assess cognitive functions linked to learning and memory. Subsequently, the animals were euthanized under chloral hydrate anesthesia, and brain tissues (prefrontal cortex and hippocampus) were dissected for biochemical analyses of TNF-α levels, acetylcholinesterase activity, and BDNF concentrations. The overall experimental design, encompassing drug delivery and cognitive testing protocols, is summarized in Figure 1.

### 2.4 Behavioral tests

For the behavioral tests, all animals were tested in a randomized order, and their activities were recorded using a video camera positioned approximately 200 cm above the center of each testing maze.

#### 2.4.1 Morris water maze test (MWM)

The Morris Water Maze (MWM) test was conducted to evaluate spatial learning and reference memory, following the methodology previously outlined (Bikri et al., 2022). The experimental setup consisted of a circular pool (120 cm in diameter × 40 cm in depth) filled with tap water maintained at 22°C to a depth of 30 cm. The MWM was conceptually divided into four quadrants (North, South, West, and East), each marked by a distinct visual cue positioned on the distal wall of the pool above the water surface. A hidden platform (11 × 14 cm) was placed in the center of one of the four quadrants, submerged 2 cm below the water surface (Figure 1), and remained in a fixed position throughout all trials. The experiment was conducted in two distinct phases. During the training phase, each subject underwent five consecutive trials, starting from different entry points around the pool while facing away from the submerged platform. Each trial provided the subject with 60 s to learn how to escape the water and locate the hidden platform. Animals that successfully reached the platform were allowed to remain on it for 10 s before being removed, whereas those failing to find the platform within 90 s were gently guided to it. In the test phase, which replicated the conditions of the training phase, the latency to reach the submerged platform was recorded using video tracking and subsequently analyzed. This latency serves as an indicator of the time required for the rat to locate the obscured, invisible platform in the opaque water, reflecting its spatial learning and memory performance.

#### 2.4.2 Recognition memory test

The Novel Object Recognition Test (NORT) is a behavioral assay designed to assess learning and memory processes in rodents, as outlined in prior research by Bikri et al. 2024 The test is conducted in an open-field arena with dimensions of 50 cm × 50 cm × 40 cm and spans three consecutive days. On the first day, rats are allowed to freely explore the arena for 5 min to acclimate to the environment. On the second day, the training phase is initiated, during which rats are exposed to two identical objects placed within the arena for 5 min, after which they are returned to their home cages. On the third day, long-term memory (LTM) is evaluated by replacing one of the familiar objects with a novel object, allowing the assessment of the rat’s ability to discriminate between the familiar and the unfamiliar item. This three-day protocol leverages the rodent’s innate cognitive capacity to recognize and differentiate objects based on prior exposure.

To evaluate short-term memory (STM), a separate trial is conducted 2 h after the 5-min training session, wherein a novel object is introduced alongside a familiar one. In contrast, long-term memory is assessed 24 h after the training phase by presenting the rat with one familiar object and one novel object distinct from those used in the STM evaluation.

Two key parameters are quantified during the NORT: (1) the preference for the novel object, calculated as the ratio of the number of investigations directed toward the novel object to the total number of investigations of both objects, and (2) exploratory behavior, defined as the duration during which the rat directs its nose toward an object at a distance of ≤3 cm without physical contact. These metrics provide insights into the rodent’s recognition memory and exploratory tendencies.

#### 2.4.3 Y-maze test

This behavior was driven by the innate curiosity of rodents to explore novel environments. Spatial working memory was assessed using the Y-maze test, as previously outlined (Bikri et al., 2022). The apparatus consists of three identical wooden arms, labeled A, B, and C (40 cm in length × 10 cm in width × 13 cm in height), arranged in a Y-shaped configuration. Each arm was painted with distinct color patterns and positioned at a 120° angle relative to the adjacent arms. The test was initiated by placing a rat at the center of the maze and allowing it to freely explore the three arms for a duration of 8 min. Spontaneous alternations, which reflect the natural propensity of rodents to seek out unexplored areas, were analyzed. This parameter was quantified as a percentage using the following formula:

$$\%\mathrm{Alternation}=\left(\mathrm{Number of Alternations}/\left[\mathrm{Total number of arm entries}–\;2\right]\right)\times 100$$

### 2.5 Preparation of tissue homogenates

Following sacrifice, the PFC and hippocampus from each cerebral hemisphere were individually and meticulously dissected. Homogenates were prepared using a Dounce homogenizer in ice-cold RIPA-based lysate buffer containing 1 mM PMSF. The homogenized samples were then subjected to centrifugation at 14,000 × g for 15 min at 4°C. The clarified supernatants were aliquoted and cryopreserved at −20°C until subsequent biochemical assays (Bikri et al., 2022).

### 2.6 Proinflammation markers measurements

The concentrations of pro-inflammatory cytokines, TNF-α and IL-6, were measured in hippocampal and PFC tissue samples using ELISA kits. Specifically, IL-6 was quantified with a kit from ThermoFisher Scientific (catalog number ERIL1B), while TNF-α was evaluated using a kit from R&D Systems (catalog number RTA00). The assays were performed in accordance with the manufacturers’ protocols, and cytokine levels were expressed as picograms per Gram of total protein.

### 2.7 Acetylcholinesterase activity

The homogenized tissue was used to determine acetylcholinesterase (AChE) activity following the method of Ellman et al. (1961). The reaction mixture contained 10 mM 5,5′-dithiobis(2-nitrobenzoic acid) (DTNB), 100 mM phosphate buffer (pH 7.5), 15 μL of supernatant, and 0.8 mM acetylthiocholine as the substrate. Absorbance was measured at 412 nm every 30 s for 2 min at 27°C. AChE activity was expressed as micromoles of acetylthiocholine hydrolyzed per hour per milligram of protein.

### 2.8 BDNF assay

BDNF levels in the prefrontal cortex and hippocampus were quantified using the ChemiKine BDNF Sandwich ELISA Kit, CYT306 (Chemicon/Millipore, Billerica, MA, United States), in accordance with the manufacturer’s instructions. The results were expressed as picograms of BDNF per milligram of total protein.

### 2.9 Statistical analysis

The data are presented as mean ± standard deviation (SD) and were analyzed using SPSS software. Statistical comparisons were conducted using one-way and two-way analysis of variance (ANOVA), followed by Tukey’s *post hoc* test for multiple comparisons. A p-value below 0.05 was deemed statistically significant.

## 3 Results

### 3.1 Subchronic effects of HgCl_2_ on fasting blood glucose in STZ-NA-induced diabetic rats

**FIGURE 2:**
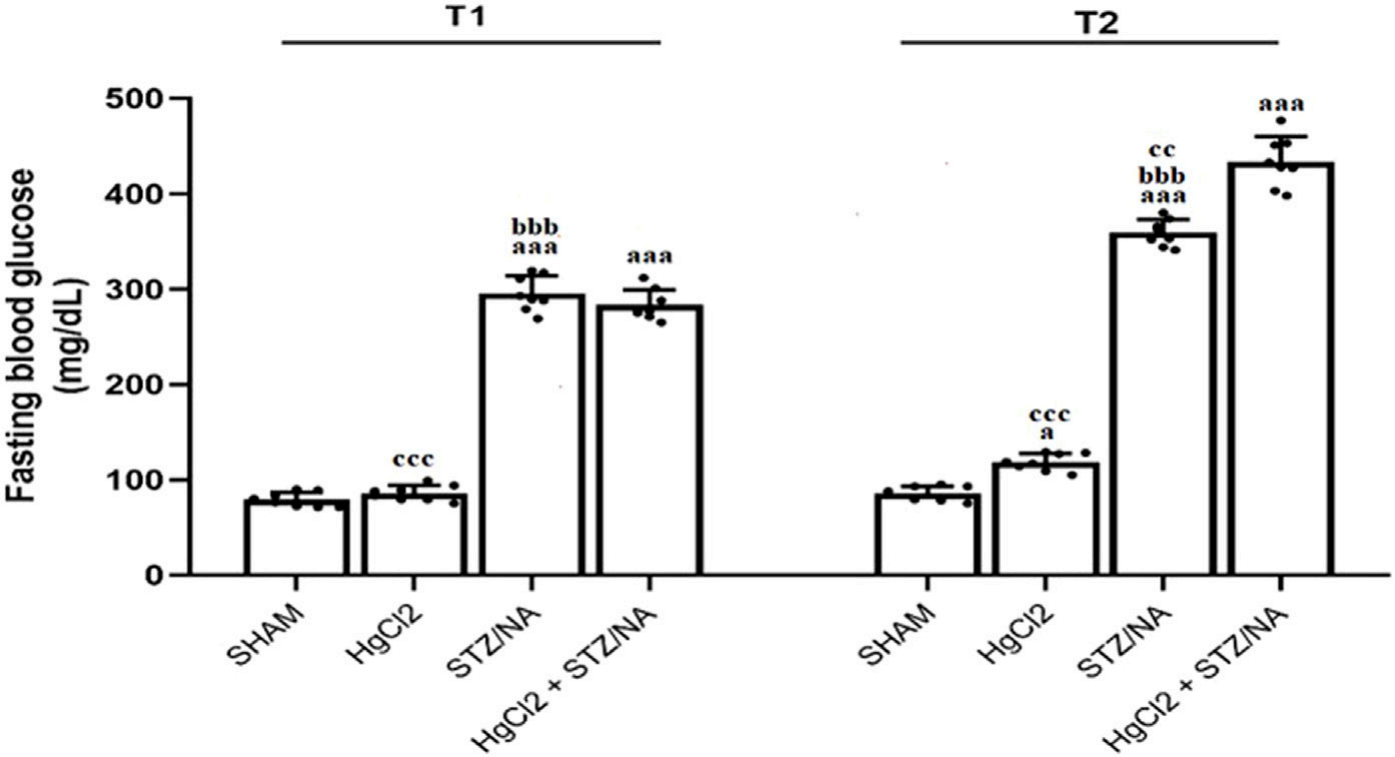
Subchronic Effects of HgCl_2_ on Fasting Blood Glucose and Body Weight in STZ-NA-Induced Diabetic Rats. All data are presented as mean ± SD (n = 8). aP < 0.05, aaP < 0.01, aaaP < 0.001 vs. Sham group; bP < 0.05 vs. HgCl2 group; cP < 0.05, CCP < 0.01, CCCP < 0.001 vs. HgCl2+STZ/NA group; ANOVA/Post hoc (Tukey test).Impact of Subchronic HgCl_2_ Exposure on Learning and Memory Functions in STZ-NA-Induced Diabetic Rats.

Figure 2 illustrates the fluctuations in fasting blood glucose (FBG) levels across the experimental groups. At T1 (72 h post-STZ/NA injection), a significant elevation in FBG levels was observed in all STZ-NA-injected rats (P = 0.000), confirming the induction of hyperglycemia compared to the Sham group. By T2 (45 days post-STZ/NA injection), both the diabetic group and the diabetic group exposed to HgCl_2_ exhibited a sustained and significant increase in FBG levels (P = 0.000). In contrast, prolonged exposure to HgCl_2_ alone for 45 days resulted in a modest but statistically significant rise in FBG levels compared to the Sham group (P = 0.041). Furthermore, the diabetic group receiving HgCl_2_ treatment for 45 days demonstrated a significantly higher FBG level (P = 0.003) compared to the untreated diabetic group, suggesting a potential exacerbation of hyperglycemia due to HgCl_2_ exposure.

**FIGURE 3:**
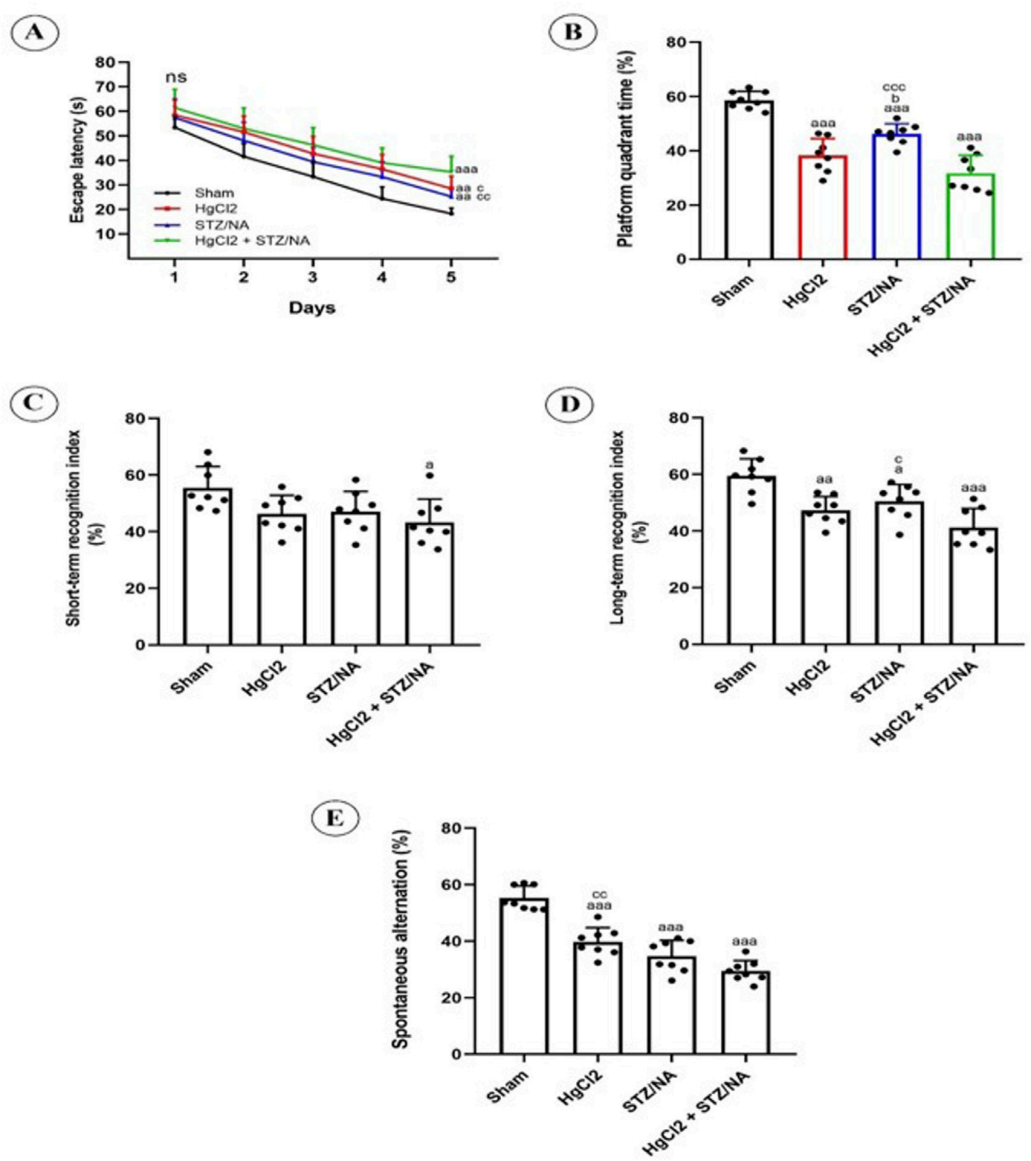
Impact of Subchronic HgCl_2_ Exposure on Learning and Memory Functions in STZ-NA-Induced Diabetic Rats. **(A)** escape latency in Morris test, **(B)** Platform quadrant time (%) in Morris test, **(C)** Short term recognition index (%) in RO test, **(D)** Long term recognition index (%) in RO test, **(E)** Spontaneous alternation (%) in Y-maze Test. All data are presented as mean ± SD (n = 8). aP < 0.05, aaP < 0.01, aaaP < 0.001 vs. Sham group; bP < 0.05 vs. HgCl2 group; cP < 0.05, CCP < 0.01, CCCP < 0.001 vs. HgCl2+STZ/NA group; ANOVA/Post hoc (Tukey test).

Spatial learning and reference memory were assessed using the Morris Water Maze (MWM) test, as shown in Figures 3A,B. Throughout the learning trials, all trained rats displayed a significant decrease in escape latency time (Figure 3A). On the final training day (day 5), there were clear differences in this time among the HgCl_2_-treated group, the diabetic group, the HgCl_2_-treated diabetic group, and the sham group (P = 0.005, P = 0.007, P = 0.000, respectively). These results suggest that the administration of HgCl_2_ over 45 days led to a marked reduction in escape latency time for the diabetic group compared to the untreated diabetic group (P = 0.008).

To assess reference memory using the MWM test, we measured the time spent in the quadrant containing the virtual platform, as shown in Figure 3B. During the probe trial, the HgCl_2_-treated group, the diabetic group, and the HgCl2-treated diabetic group spent significantly less time in the virtual platform quadrant compared to the sham group (P = 0.000). Notably, subchronic administration of HgCl_2_ to the diabetic group resulted in a more pronounced reduction in time spent in this quadrant compared to the untreated diabetic group (P = 0.000). Additionally, the results indicated that administering HgCl_2_ to normoglycemic rats for 45 days significantly decreased the time spent in the virtual platform quadrant when compared to diabetic rats (P = 0.031).

The recognition index percentages during the STM and LTM phases revealed significant differences among the groups, as shown in Figures 3C,D. Specifically, the HgCl_2_-treated diabetic group showed a notable decrease in STM (Figure 3C) compared to the sham group (P = 0.031). In contrast, the HgCl_2_-treated, diabetic, and HgCl_2_-treated diabetic groups all exhibited significant reductions in the long-term recognition memory index compared to the sham group (P = 0.006, P = 0.019, P = 0.000, respectively). Additionally, the combination of hyperglycemia and 45 days of subchronic HgCl_2_ administration led to a significant decline in long-term recognition memory compared to the untreated diabetic group (P = 0.030).

In this study, the Y-maze test was employed to evaluate Spatial Working Memory abilities, as shown in Figure 3E. The results demonstrated a significant reduction in the percentage of correct alternations among the HgCl_2_-treated group, the diabetic group, and the HgCl_2_-treated diabetic group compared to the sham group (P = 0.000). Furthermore, the combination of hyperglycemia and subchronic HgCl_2_ administration over 45 days led to a notable decrease in the percentage of correct alternations when compared to the normoglycemic HgCl_2_-treated group (P = 0.0044).

### 3.2 Effects of subchronic HgCl_2_ exposure on hippocampal and prefrontal cortex TNF-alpha and IL-6 levels in diabetic rats

**FIGURE 4:**
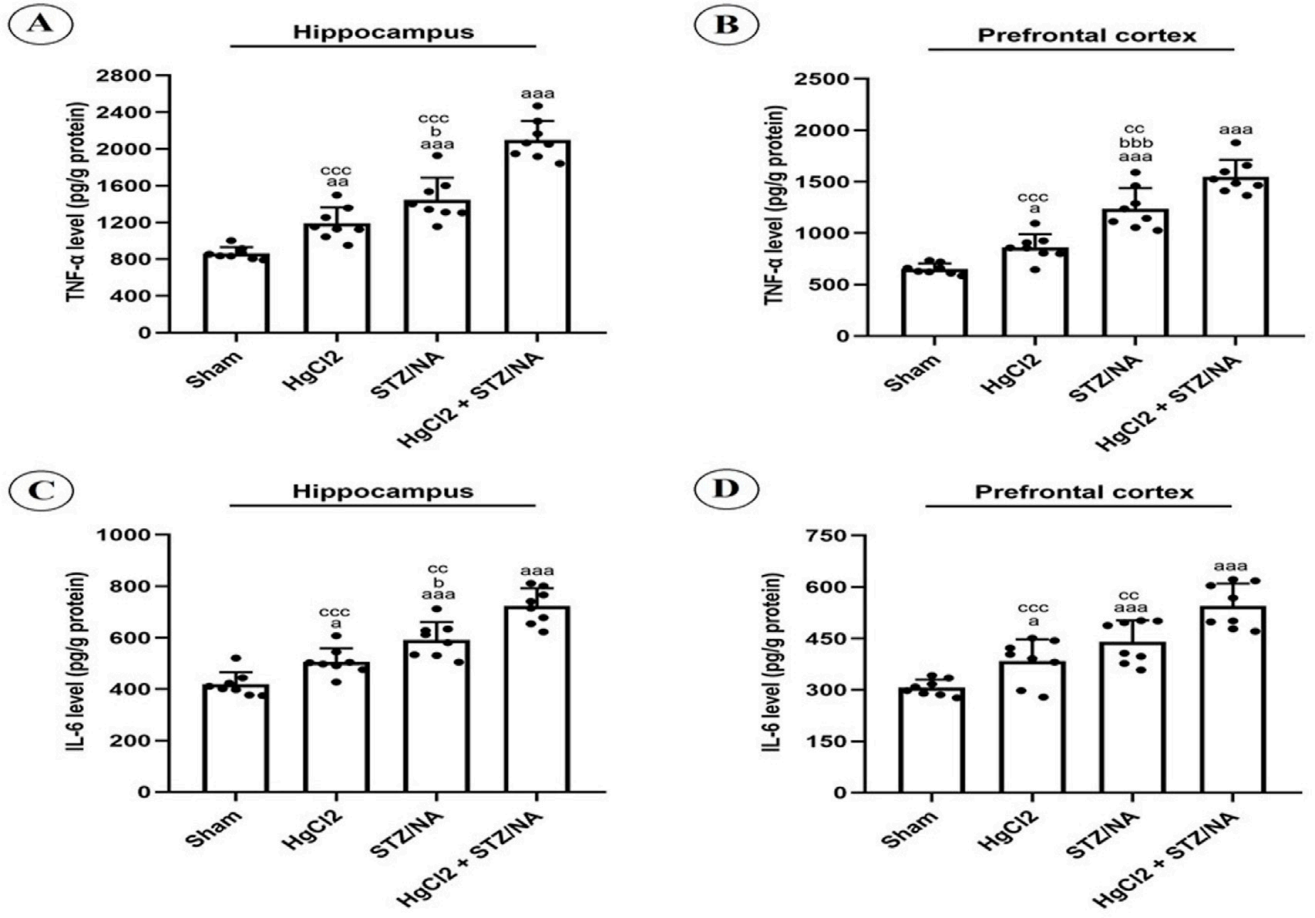
Effects of Subchronic HgCl_2_ Exposure on TNF-alpha and IL-6 Levels in the Hippocampus and Prefrontal Cortex of Diabetic Rats. **(A)** TNF-alpha level in the hippocampus, **(B)** TNF-alpha level in the PFC, **(C)** IL-6 level in the hippocampus, **(D)** IL-6 level in the PFC. All data are presented as mean ± SD (n = 8). aP < 0.05, aaaP < 0.001 vs. Sham group; bP < 0.05 vs. HgCl2 group; CCP < 0.01, CCCP < 0.001 vs. HgCl2+STZ/NA group; ANOVA/Post hoc (Tukey test).

To assess the level of neuroinflammation in diabetic rats treated or untreated with HgCl_2_, we measured the levels of TNF-alpha and IL-6 in the hippocampus and prefrontal cortex, as illustrated in Figure 4. Statistical analysis revealed a significant increase in TNF-alpha (P = 0.008) and IL-6 (P = 0.025) levels in the hippocampus, as well as TNF-alpha (P = 0.038) and IL-6 (P = 0.048) levels in the prefrontal cortex of normoglycemic rats treated for 45 days with HgCl_2_ compared to the sham group. This increase was highly significant in both tissues in diabetic rats and diabetic rats treated with HgCl_2_ compared to the sham group (P = 0.000). Additionally, the results showed that TNF-alpha (P = 0.043) and IL-6 (P = 0.046) levels were significantly higher in the hippocampus, and for TNF-alpha (P = 0.000) and IL-6 (P = 0.199) in the prefrontal cortex of diabetic rats compared to non-diabetic rats treated with HgCl_2_. Furthermore, the combination of diabetes and HgCl_2_ for 45 days significantly increased TNF-alpha levels in both the hippocampus and prefrontal cortex compared to normoglycemic rats treated with HgCl_2_ (P = 0.000 for both) and untreated diabetic rats (P = 0.000 and P = 0.007, respectively). Similar results were observed for IL-6 levels in the hippocampus and prefrontal cortex compared to normoglycemic rats treated with HgCl_2_ (P = 0.000 for both) and untreated diabetic rats (P = 0.004 and P = 0.005, respectively).

### 3.3 Effects of subchronic HgCl_2_ Exposure on Acetylcholinesterase Activity in the hippocampus and prefrontal cortex of STZ-NA-induced diabetic rats

**FIGURE 5:**
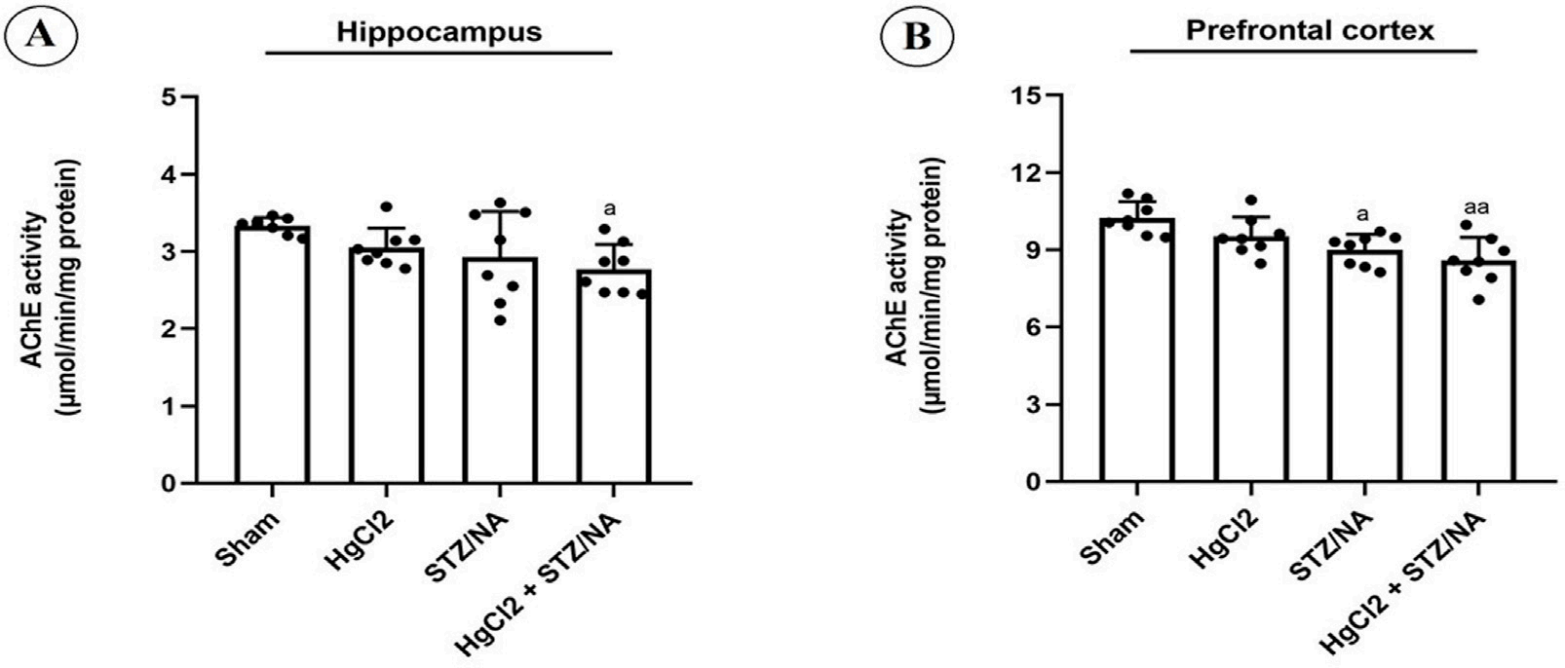
Effects of Subchronic HgCl_2_ Exposure on Acetylcholinesterase Activity in the Hippocampus and Prefrontal Cortex of STZ-NA-Induced Diabetic Rats. **(A)** Acetylcholinesterase activity in the Hippocampus, **(B)** Acetylcholinesterase activity in the PFC. All data are presented as mean ± SD (n = 8). aP < 0.05, aaP < 0.01, ANOVA/Post hoc (Tukey test).

In the present study, the activity of AChE in the hippocampus and prefrontal cortex was assessed, with results presented in Figure 5. Subchronic administration of HgCl_2_ for 45 days in normoglycemic rats did not induce a significant alteration in AChE activity in either the hippocampus (P = 0.404) or the prefrontal cortex (P = 0.227) compared to the sham group. However, hyperglycemia for 45 days significantly decreased AChE activity in the prefrontal cortex (P = 0.012) compared to the sham group. Furthermore, the results indicated a significant reduction in AChE activity in the hippocampus (P = 0.020) and prefrontal cortex (P = 0.001) in diabetic rats treated with HgCl_2_ compared to the sham group. Conversely, no significant alterations in AChE activity were observed in the hippocampus or prefrontal cortex among the three experimental groups (P > 0.05).

### 3.4 Outcomes of subchronic HgCl_2_ exposure on BDNF levels in the hippocampus and prefrontal cortex of STZ-NA-induced diabetic rats

**FIGURE 6:**
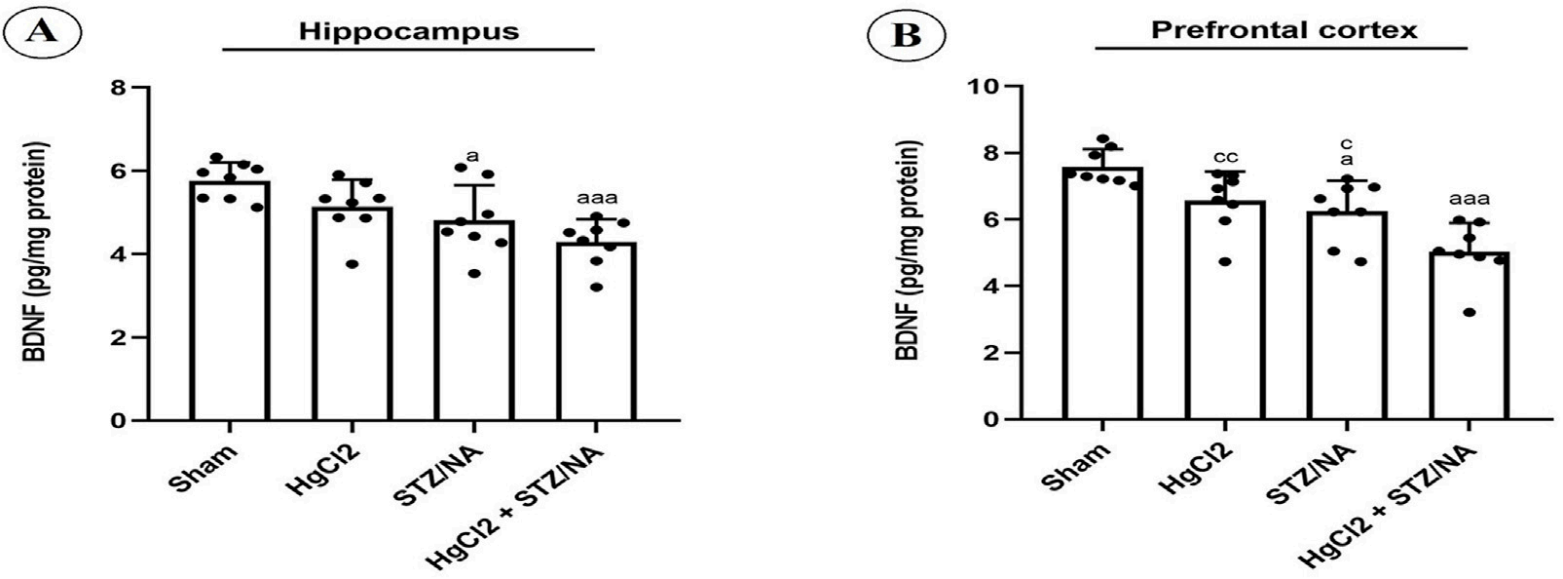
Effects of Subchronic HgCl_2_ Exposure on BDNF Levels in the Hippocampus and Prefrontal Cortex of STZ-NA-Induced Diabetic Rats. **(A)** Hippocampal BDNF level, **(B)** BDNF level in the PFC. All data are presented as mean ± SD (n = 8). aP < 0.05, aaaP < 0.001 vs. Sham group; cP < 0.05, CCP < 0.01 vs. HgCl2+STZ/NA group; ANOVA/Post hoc (Tukey test).

This investigation focused on assessing BDNF levels in the hippocampus and prefrontal cortex, with findings illustrated in Figure 6. Subchronic administration of HgCl_2_ for 45 days in normoglycemic rats did not result in significant alterations in BDNF levels in either the hippocampus (P = 0.221) or the prefrontal cortex (P = 0.081) compared to the sham group. Nevertheless, persistent hyperglycemia for 45 days significantly reduced BDNF levels in both the prefrontal cortex (P = 0.014) and the hippocampus (P = 0.030) compared to the sham group. Furthermore, a highly significant decrease in BDNF levels was observed in both the hippocampus and prefrontal cortex in diabetic rats treated with HgCl_2_ (P = 0.000 for both) compared to the sham group. Conversely, no significant differences in BDNF levels were noted between the diabetic group and the normoglycemic group treated with HgCl_2_ (P > 0.05). Notably, a significant difference was observed in the prefrontal cortex of diabetic rats treated with HgCl_2_ compared to both untreated diabetic rats (P = 0.026) and normoglycemic rats treated with HgCl_2_ (P = 0.007).

## 4 Discussion

The present study rigorously investigated the subchronic effects of HgCl_2_ exposure on various pathophysiological parameters in STZ-NA-induced diabetic rats. Our findings reveal remarkable disturbances in FBG levels, pronounced CI, and notable alterations in key inflammatory cytokines, including TNF-alpha and IL-6. Furthermore, we observed changes in AChE activity and BDNF levels within critical brain regions such as the hippocampus and PFA. These results emphasize the substantial impact of HgCl_2_ exposure on both metabolic and neurological functions, highlighting its detrimental effects, particularly in the context of hyperglycemia.

This study finding revealed a significant elevation in FBG levels in diabetic rats exposed to HgCl_2_. This observation is consistent with existing literature, which links heavy metal exposure, particularly mercury, with disruptions in glucose homeostasis (Barnes and Kircher, 2005). These disturbances are likely mediated by inflammation/oxidative stress, which adversely affects pancreatic function and promotes insulin resistance (Chen et al., 2012).

Additionally, our study demonstrates that HgCl_2_ exposure for 45 days significantly exacerbates neuroinflammation in diabetic rats, as evidenced by increased levels of TNF-α and IL-6 in the PFA and hippocampus. The inflammatory response was notably more pronounced in diabetic rats compared to normoglycemic rats, suggesting that HgCl_2_ potentiates hyperglycemia-induced neuroinflammation. This rise in inflammatory cytokines is in line with the findings of Li et al. (2022), who showed that HgCl_2_ activates the NF-κB pathway, leading to an upregulation of pro-inflammatory cytokines like TNF-α and IL-6 in the brain. The activation of NF-κB is a key driver of neuroinflammatory processes and neuronal dysfunction (Anilkumar and Wright-Jin, 2024). In this line, several studies have underscored the role of NF-κB activation in the pathophysiology of diabetes and its complications (Patel and Santani, 2009). Specifically, hyperglycemia triggers NF-κB activation, which stimulates the production of inflammatory cytokines that contribute to diabetic complications (Taïlé et al., 2021), including neuronal damage. As highlighted by Patel and Santani (2009), hyperglycemia in diabetes activates a cascade of molecular events that result in diabetic neuropathy, with NF-κB playing a central role. Our findings further support this model, demonstrating that HgCl_2_ exposure, in conjunction with diabetes, significantly enhances TNF-α and IL-6 levels in both the hippocampus and PFC when compared to diabetic rats without HgCl_2_ treatment. This reinforces the concept that HgCl_2_ potentiates neuroinflammation in the diabetic brain.

Several studies have independently demonstrated the pro-inflammatory effects of hyperglycemia and HgCl_2_. However, our study is the first to reveal that the simultaneous occurrence of these two pathological conditions leads to a synergistic exacerbation of inflammatory complications, surpassing the impact observed with each factor alone. This finding highlights the potential for increased vulnerability in individuals exposed to both metabolic dysregulation and heavy metal toxicity, underscoring the need for further investigation into their combined pathophysiological mechanisms.

Building upon the observed dysregulation of pro-inflammatory cytokines, our findings further demonstrate that subchronic HgCl_2_ exposure exacerbates cognitive deficits in STZ-NA-induced diabetic rats. The relationship between systemic inflammation and neurological impairment is well-documented, as neuroinflammation disrupts crucial processes for memory consolidation and retrieval, including synaptic plasticity (Golia et al., 2019) and hippocampal neurogenesis (Chesnokova et al., 2016). Elevated levels of TNF-α and IL-6, as reported in our study, are known to compromise the BBB and activate microglial cells, leading to increased oxidative stress and neuronal apoptosis (Rochfort et al., 2016; Goshi et al., 2025). This inflammatory environment likely contributes to the cognitive deterioration observed in the HgCl_2_-treated diabetic group.

Our study further confirms that subchronic exposure to HgCl_2_ exacerbates cognitive deficits in diabetic rats, particularly impairing spatial learning, working memory, and both short- and long-term recognition memory. The combination of hyperglycemia and HgCl_2_ exposure produces synergistic neurotoxic effects, leading to a significantly greater CI compared to either condition alone. These findings align with previous studies showing that both hyperglycemia and HgCl_2_ exposure have a significant detrimental impact on cognitive functions (Asuku et al., 2024; Kassab et al., 2019). Notably, this is the first study to evaluate the synergistic interaction between hyperglycemia and HgCl_2_, which further exacerbates these cognitive deficits and underscores the heightened neurotoxic risk in conditions involving metabolic disturbances. Chronic exposure to inorganic mercury has been linked to deficits in working memory and episodic memory, likely due to its neurotoxic effects on the hippocampus and prefrontal cortex. These brain regions are essential for encoding, consolidating, and retrieving memories. Inorganic mercury disrupts synaptic plasticity by interfering with glutamate and calcium signaling pathways, impairing neuronal communication necessary for memory formation (Farina et al., 2011). Additionally, HgCl-induced oxidative stress and mitochondrial dysfunction exacerbate neurodegeneration, further compromising cognitive performance (Bjørklund et al., 2017). Epidemiological studies of populations exposed to mercury through occupational or environmental sources (e.g., mining, contaminated fish consumption) report poorer performance in memory-related tasks, such as delayed recall and spatial navigation, compared to unexposed groups (ATSDR, 2022). These results highlight the critical need for monitoring HgCl exposure levels and implementing protective measures for vulnerable populations, particularly individuals with diabetes, who may face compounded risks due to pre-existing oxidative stress and metabolic impairments that synergize with mercury’s neurotoxic effects.

In the Morris Water Maze test, HgCl_2_-treated diabetic rats exhibited severe deficits in spatial learning and reference memory, as evidenced by significantly prolonged escape latencies and reduced time spent in the target quadrant during the probe trial. These results are consistent with the findings of Bikri et al. (2024), who revealed that hyperglycemia impairs spatial learning and reference memory, which is linked to neuroinflammation in the hippocampus and prefrontal cortex. Additionally, Behzadfar et al. (2020) showed that HgCl_2_ exposure adversely affects spatial learning, reference memory, and mitochondrial function in the hippocampus of rats.

Furthermore, both short- and long-term recognition memory were significantly impaired in HgCl_2_-treated diabetic rats, as indicated by notably reduced recognition indices for both STM and LTM. These results suggest early and persistent deficits in memory consolidation. The more pronounced decline in LTM compared to STM indicates that prolonged exposure to HgCl_2_ and hyperglycemia disrupts the stabilization of memory, reflecting the cumulative burden of metabolic and toxic stressors on cognitive networks. Supporting this, Dinel et al. (2011) found that recognition memory in diabetic rats and mice was impaired, which was associated with elevated levels of pro-inflammatory cytokines (IL-6 and TNF-α), suggesting a link between inflammation and memory deficits. Recent research has also shown that hyperglycemia in type 2 diabetes significantly affects both STM and LTM, accompanied by altered AChE activity and significant inflammatory responses in the hippocampus and prefrontal cortex (ElKouch et al., 2025). In addition, a study by Mello-Carpes et al. (2013) revealed that chronic exposure to low concentrations of HgCl_2_ induces deficits in object recognition and aversive memory in rats, further emphasizing the neurotoxic effects of HgCl_2_ on cognitive functions.

Moreover, spatial working memory, assessed via the Y-maze test, was similarly impaired. HgCl_2_-treated diabetic rats exhibited impaired alternation performance compared to the normoglycemic HgCl_2_-treated group, highlighting deficits in working memory likely linked to disrupted fronto-hippocampal circuitry. CI commonly observed in patients with type 2 diabetes include a decline in working memory (Chen et al., 2014). Furthermore, Liao et al. (2025) synthesized findings suggesting that type 2 diabetes promotes working memory decline associated with biochemical dysfunction. In an experimental study conducted by Azirar et al. (2025), HgCl_2_ exposure was shown to impair working memory in rats, an effect that was associated with a disruption of central antioxidant responses.

Cognitive decline in type 2 diabetes and heavy metal exposure is closely linked to disruptions in cholinergic neurotransmission and neurotrophic support, particularly through alterations in AChE activity and BDNF levels (Davarpanah et al., 2021; Mushtaq et al., 2014; Frasco et al., 2007). Dysregulation of these two markers has been observed in various neurodegenerative and metabolic disorders, highlighting their importance in cognitive function. In diabetes, chronic hyperglycemia induces oxidative stress and neuroinflammation, leading to a reduction in BDNF expression (Davarpanah et al., 2021) and alterations in AChE activity (Mushtaq et al., 2014), both of which contribute to memory deficits and impaired executive function. Similarly, mercury exposure disrupts cholinergic signalling (Frasco et al., 2007) and neurotrophic factor regulation (Li et al., 2022), exacerbating neuronal damage and CI. Given this, our study sought to investigate how subchronic HgCl_2_ exposure interacts with type 2 diabetes to influence AChE activity and BDNF levels, potentially aggravating cognitive deficits.

Our findings indicate that AChE activity is significantly reduced in the hippocampus and PFA of diabetic rats, an effect that is further exacerbated by HgCl_2_ exposure. This suggests a combined neurotoxic impact of hyperglycemia and mercury on cholinergic neurotransmission. Reduced AChE activity in diabetes has been previously reported, with studies indicating that hyperglycemia disrupts ACh metabolism, leading to synaptic dysfunction and CI (Mushtaq et al., 2014). The decline in AChE activity observed in our diabetic rats aligns with findings where decreased AChE correlated with deficits in memory functions (Haam and Yakel, 2017). The additional inhibition of AChE in diabetic rats treated with HgCl_2_ suggests that mercury exacerbates cholinergic dysfunction. Prior research has shown that choline acetyltransferase, a key enzyme responsible for ACh synthesis, is inhibited by mercury exposure, further exacerbating ACh deficiency and potentially worsening CI (Althobaiti, 2025). The disruption of cholinergic signaling is further compounded by the role of AChE, an enzyme responsible for ACh hydrolysis at synaptic clefts. This mechanism may explain the aggravated CI observed in our diabetic HgCl_2_-exposed rats.

Disruptions in the function of certain neurotransmitters, such as AChE, combined with reduced release of BDNF in type 2 diabetes mellitus, significantly heighten the risk of CI in affected individuals (Ahmad et al., 2022). BDNF, a crucial neurotrophin, plays an essential role in neuronal differentiation, survival, and synaptic plasticity, all of which are vital for memory formation and learning processes (Bikri et al., 2024). It is predominantly expressed in brain regions associated with higher cognitive functions, including the hippocampus, and cortex, where it supports synapse formation and regulates neurotransmission (Nemati et al., 2025). BDNF plays a significant role in cognitive health, with its signaling pathway contributing to reduced apoptosis in hippocampal neurons and protection against hippocampal atrophy, a key feature of CI (Peritore et al., 2020). In this context, our study revealed a significant reduction in BDNF levels in both the hippocampus and PFA of diabetic rats, with a more pronounced decrease in those exposed to HgCl_2_. In agreement with these findings, Kaviarasana et al. (2015) reported a notable decline in BDNF levels among diabetic patients compared to healthy individuals. Furthermore, they identified a positive correlation between BDNF and interleukin-10, suggesting that these biomarkers may be involved in disease progression. These results further support the hypothesis that decreased BDNF levels could contribute to central inflammation associated with hyperglycemia. In addition, Tang et al. (2017) demonstrated that diabetic control rats showed reduced expression of BDNF and the cAMP-response element binding protein gene in the hippocampus, accompanied by impairments in learning and memory compared to normal control rats. Along these lines, recent studies have indicated that BDNF overexpression can reduce inflammation in the hippocampus of diabetic mice. These findings suggest that specific inflammatory signaling pathways may play a central role in mediating the neuroinflammation alleviation by BDNF in diabetic mice. This supports the potential of enhancing BDNF expression as a therapeutic strategy to mitigate diabetes-associated neuroinflammation (Han et al., 2019). On the other hand, HgCl_2_ exposure appears to exacerbate this effect, as evidenced by our study, which showed a further decline in BDNF levels in diabetic rats exposed to HgCl_2_. This observation aligns with previous experimental findings, where HgCl_2_-induced brain damage was associated with altered BDNF expression and the inhibition of NF-κB signaling pathways (Li et al., 2022). Furthermore, an experimental study has shown that, despite its low liposolubility, inorganic mercury can cause significant damage to the central nervous system. Prolonged exposure to low doses in rats leads to considerable CI and hippocampal damage, underscoring the neurotoxic potential of even minimal mercury exposure over time (Aragão et al., 2018).

In conclusion, our findings indicate that the combined effects of hyperglycemia and HgCl_2_ exposure disrupt cholinergic function and neurotrophic support, leading to cognitive decline. These alterations appear to be driven by neuroinflammation in both the prefrontal cortex and hippocampus. Notably, the pronounced reduction of BDNF in these regions suggests that they may be particularly vulnerable to both neurotoxic and metabolic insults. To address these issues, future research should explore potential neuroprotective strategies, such as antioxidant or anti-inflammatory interventions, to mitigate CI in diabetic conditions exacerbated by environmental neurotoxins, such as HgCl_2_.

## Data Availability

The raw data supporting the conclusions of this article will be made available by the authors, without undue reservation.
